# Population Size Estimation From Capture-Recapture Studies Using shinyrecap: Design and Implementation of a Web-Based Graphical User Interface

**DOI:** 10.2196/32645

**Published:** 2022-04-26

**Authors:** Anne F McIntyre, Ian E Fellows, Steve Gutreuter, Wolfgang Hladik

**Affiliations:** 1 Division of Global HIV & TB Center for Global Health Centers for Disease Control and Prevention Atlanta, GA United States; 2 Fellows Statistics San Diego, CA United States

**Keywords:** population size estimation, multiple-source capture-recapture, Bayesian models, latent-class models, Shiny, HIV, key populations, epidemiology, digital health, online health application, populations, risk factors, online communities

## Abstract

**Background:**

Population size estimates (PSE) provide critical information in determining resource allocation for HIV services geared toward those at high risk of HIV, including female sex workers, men who have sex with men, and people who inject drugs. Capture-recapture (CRC) is often used to estimate the size of these often-hidden populations. Compared with the commonly used 2-source CRC, CRC relying on 3 (or more) samples (3S-CRC) can provide more robust PSE but involve far more complex statistical analysis.

**Objective:**

This study aims to design and describe the Shiny application (shinyrecap), a user-friendly interface that can be used by field epidemiologists to produce PSE.

**Methods:**

shinyrecap is built on the Shiny web application framework for R. This allows it to seamlessly integrate with the sophisticated CRC statistical packages (eg, Rcapture, dga, LCMCR). Additionally, the application may be accessed online or run locally on the user’s machine.

**Results:**

The application enables users to engage in sample size calculation based on a simulation framework. It assists in the proper formatting of collected data by providing a tool to convert commonly used formats to that used by the analysis software. A wide variety of methodologies are supported by the analysis tool, including log-linear, Bayesian model averaging, and Bayesian latent class models. For each methodology, diagnostics and model checking interfaces are provided.

**Conclusions:**

Through a use case, we demonstrated the broad utility of this powerful tool with 3S-CRC data to produce PSE for female sex workers in a subnational unit of a country in sub-Saharan Africa.

## Introduction

### Background

Accurate knowledge of population size is critical in many areas of science but a challenge whenever complete counts are too difficult or expensive to be obtained. One such area is the HIV pandemic, which increasingly is driven by high-risk behaviors that define “key populations” (KP), among them, female sex workers (FSW), men who have sex with men (MSM), and people who inject drugs (PWID) [1]. Global, national, and local HIV control efforts all require knowing the size of these high-risk populations to monitor the epidemic in terms of density and distribution of populations over time and to inform effective and appropriately scaled program development, target setting, and resource allocation. Yet, there is no gold standard to derive reliable population size estimates (PSE). Instead, public health teams and stakeholders use a wide range of methods, many of which are not based on empirical data nor sound statistical concepts [2,3], potentially producing poor-quality estimates. Estimates of population sizes derived from programmatic mapping [4,5] enumerate members of the population attending venues during the exercise but often fail to account for the less socially visible, resulting in underestimates. Other nonempirical subjective methods such as Wisdom of the Crowd [6,7] and the Delphi methods [3,8] are susceptible to bias and the influence of individuals.

Capture-recapture (CRC) globally has seen wide use for PSE, including for the HIV pandemic [9-18]. The basic idea behind CRC is to engage in 2 or more encounter events or sources (these might also be referred to as samples, captures, or lists), recording which individuals appear in which events and relating the number of individuals sampled once to those sampled repeatedly. Most CRC exercises include 2 encounter events with the key assumption being that the 2 samples (2S) are independent [19]. Unfortunately, many such 2S-CRC exercises may suffer from violating this assumption resulting in overestimates (negative dependence between 2 samples) or underestimates (positive dependence between 2 samples) [3,19,20]. CRC with 3 (or more) samples (3S-CRC) relaxes this assumption, as interaction terms may be added to the statistical models to address source dependencies. Given sufficient overlap of samples and independence of samples, 3S-CRC allows for more sophisticated analysis compared with 2S-CRC [18,21], resulting in more accurate PSE. Statistical support for these analyses might not be available, creating a critical challenge for field epidemiologists to produce robust PSE.

Several statistical models satisfy the requirements to perform the aforementioned sophisticated analysis of 3S-CRC data: log-linear models, Bayesian model averaging, and Bayesian nonparametric latent-class models. Log-linear models are a classic methodology for the analysis of multiple source CRC data. Variants are implemented that allow for varying capture probabilities across events and heterogeneous capture probabilities among members of the population. Bayesian model averaging allows the analyst to flexibly account for list dependency by creating models for all possible dependencies and averaging over them in a way that is proportional to the probability that the dependence is correct. The Bayesian latent class model deals with heterogeneity in a novel way. It posits that there are unobserved subgroups in the data with different capture probabilities for each capture event. The number of these groups and their probabilities are unknown. The algorithm uses a Bayesian framework to estimate these, along with the population size. Application of these 3 types of statistical models requires computational expertise. This is a barrier to the use of CRC involving 3 or more sources, as it typically requires knowledge of specialized software [22] or programming in languages such as R [23]. To fill this need, we present a graphical user interface, shinyrecap, that guides the user through sample size estimation, data preparation and exploration, and PSE using CRC studies.

### Objectives

The objectives of this paper were to describe *shinyrecap,* a free, web-based application facilitating the format and analysis of CRC data for PSE.

## Methods

### Overview of the Capture-Recapture Method

The application of ratio estimation for PSE from multiple encounters dates to at least 1787 [24] and gained popularity primarily among animal ecologists more than a century later [25-27], although applications abound in other areas, including epidemiology [28,29]. Early applications were restricted to sampling on 2 occasions or from 2 lists, wherein individuals encountered during the first survey are offered an identifying mark. For KP CRC, these identifiers are inexpensive but memorable unique objects or “gifts” such as brightly colored rubber bracelets or distinctive key chains. The number of individuals who accept the unique gifts are documented. The same process is repeated during a second survey, during which individuals are also asked about having received a gift during the previous capture. Estimation of the unknown number of population members from 2 samples requires the strong assumptions that (1) the population is static over the sampling interval, (2) the identifying unique objects or gifts are not lost nor misidentified, (3) individuals are sampled independently during the surveys (list independence), and (4) every population member shares a common and constant probability of encounter during the surveys (homogeneity). The first assumption is well-approximated by sampling over short time intervals. However, the remaining assumptions are unlikely to hold.

The next major innovation was the extension of estimation to data collected from 3 or more samples [30,31]. This enables relaxation of the third and fourth assumptions using statistical models that account for sampling dependence and various forms of inhomogeneity (ie, nonuniform) in encounter probabilities [27,30,32-35]. To understand why more samples allows for the assumption relaxation, consider a 3S-CRC where each capture is the same size. If the population is homogeneous and all individuals have the same probability of being captured in each sample (p_1_), then the probability of being captured in all 3 samples would simply be p_1_^3^. On the other hand, if half the population has a capture probability of p_1_ and the other half has probability p_2_, then the probability of a random person being captured in all 3 would be 0.5(p_1_^3^ + p_2_^3^). By comparing the counts of individuals captured in all 3 samples to what would be expected if there was homogeneity, we can measure and model it. Log-linear models, Bayesian model averaging, and Bayesian Dirichlet process mixture models (nonparametric latent-class models) and each model heterogeneity in different ways, allowing for the production of more accurate estimates in the presence of inhomogeneity.

### Overview of Relevant Statistical Models

#### Log-Linear Models

Models for capture probabilities originated in the discipline of animal ecology [27,34]. The natural logs are modeled as linear combinations of factors representing various forms on inhomogeneity. Four general classes of models are produced, representing a wide range of model complexity: Captures have the same probability, and individuals are uniform (M_0_); captures might have different probabilities, and individuals are uniform (M_t_); captures have the same probability, and individuals may be heterogeneous (M_h_); and captures may have different probabilities, and individuals may be heterogeneous (M_th_). Selection of a single “best” model is typically done using either the Akaike or Bayesian information criterion (AIC and BIC, respectively) [36]. For these, lower values indicate a “better” model fit.

For heterogeneous models, log-linear models require the specification of a parametric distribution for the population’s log odds of being captured. These are typically set to be either Normal, Poisson, Gamma, or Darroch. Additionally, the Chao (lower bound) correction can be used to obtain a lower bound on the population size rather than an estimate of it.

The “Normal” model incorporates heterogeneity as a Gaussian mixing distribution [37]. The Poisson, Darroch, and Gamma options incorporate different heterogeneity correction columns into the design matrix. The Darroch, and especially the Gamma, correction may produce distinctly large heterogeneity corrections and estimates of population size. Unfortunately, the correct model specifications are frequently not identifiable (roughly, parameters are not informed by the data), and so choosing based on any information criteria can lead to misspecified models [38].

#### Bayesian Model Averaging of Log-Linear Models

Bayesian model averaging is geared to be robust to list dependence. Ideally, one would like to have all capture events be independent draws from the population. In many cases, however, some capture events may be related. For example, in a citywide survey of PWID, it might happen that the first 2 capture events were more heavily concentrated in one area of the city than the third event, introducing potential dependence. When list dependence is present, the interactions between events should be considered.

The natural logs of expected frequencies of observable encounter combinations can be modeled as linear combinations of main and interaction effects of the sampling events [32,35]. This allows the model to flexibly account for list dependence among the various samples. Bayesian model averaging enumerates all possible models of list dependency and then puts a prior on the likelihood that each model is the true one, with more complex models typically having lower prior probability than less complex models. Combining this prior with a prior for population size allows one to calculate a posterior estimate of population size averaging over all possible models. In this posterior, estimates from each model are weighted by the posterior probability of the model, yielding a single estimate that includes model uncertainty. Some form of model averaging is important given that there may be limited information in the data available to identify the true model out of the large number of potential models [22].

The first step in the analysis is to specify a prior distribution for population size. This represents the analyst’s prior knowledge about population size along with uncertainty. By default, a “noninformative” improper prior is used, which is proportional to 1 divided by the population size. Typically, analysts will have access to at least a rough idea of the range of possible population sizes from previous PSE reports or literature reviews. This information can be incorporated into the prior parameterized as a log-normal distribution with a truncation at a specified maximum population size. The “delta” parameter controls the prior, favoring simple models in the model averaging. This parameter is more difficult to interpret, and it is set to 2^–^*^k^* by default, where *k* is the number of encounter events. Lower values indicate less prior weight on more complex list interactions. Once the prior is specified, the posterior probability distribution of the population size can be calculated.

#### Bayesian Nonparametric Latent-Class Models

Instead of assuming a parametric probability function for capture probability, as is done by traditional log-linear models, this approach posits that the population is divided into a number of groups, with members in each group having the same homogeneous capture probability. The number of homogeneous strata in a population is uncertain, and covariates that identify those classes may not be available. Thus, the strata are said to be latent, and strata identities are treated as missing data. Estimation is naturally accomplished using mixtures of distributions. A clever implementation of Bayesian nonparametric latent-class modeling can then be used to estimate population size [21]. Both the number of strata and the strata capture probabilities are inferred via Bayesian inference, with a stick-breaking Dirichlet process prior enforcing model parsimony such that models with fewer latent strata are preferred.

The degree to which fewer strata are preferred is controlled by a prior on the stick-breaking process parameterized as a Gamma distribution with shape and scale values. The relationship between the Gamma distribution and the number of latent groups is complex and mediated by a stick-breaking process. In general, the default values of 0.25 for both the shape and scale parameters result in a reasonably diffuse prior.

Estimation is based on the posterior distribution of population size, of which a sample is constructed using Markov chain Monte Carlo (MCMC) simulation. MCMC algorithms start from initial values and produce serially correlated “chains” of samples from some distribution. That distribution converges to the joint posterior distribution only after some potentially large number of “burn-in” iterations. Therefore, valid inferences can be made only after discarding the burn-in iterations.

### shinyrecap Application User Interface

*shinyrecap* was developed using the Shiny [23] web framework for R [39]. Shiny is a flexible, open-source toolkit used to build web applications with rich interactivity that can easily produce tables, visualize data, and create dashboards. The advantage of this framework is that it makes it easy to expose advanced algorithms and packages written in R to a noncoding audience. In *shinyrecap*, we leveraged the algorithms from the *Rcapture* [40] package for log-linear modeling, the *dga* package for Bayesian model averaging [41], and the *LCMCR* [21] package for Bayesian latent-class modeling. Whereas it would normally take substantial experience with R to use those packages, *shinyrecap* provides easy access to a wider audience with “the click of a button.”

*shinyrecap* has been made available for public use [42] and does not require installation of or experience with R. Client-server communication occurs over a secure-sockets layer (SSL) protocol connection. Required data inputs are minimal and can be aggregate or individual-level. Any data provided to *shinyrecap* persist only for the session; neither input nor output data are saved on the web server. This provides users with security protection against third-party traffic analysis and any security intrusions into the server not concurrent with the user’s session. *shinyrecap* offers a tutorial video and manual, and help buttons are presented where input information is required in each *shinyrecap* module.

Alternatively, R users can launch the interface locally from any computer by entering the following into the R console:



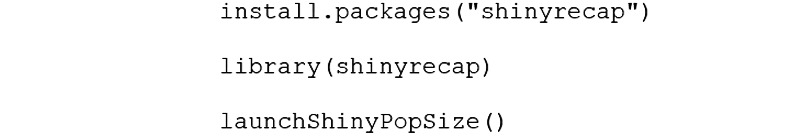



*shinyrecap* is structured in 3 parts. First, it supports the design of CRC studies by providing a tool for sample size estimation. Second, it provides a data formatting tool to assist with the data processing of CRC surveys. Finally, it provides the analysis tool to generate the estimates and outputs required for PSE.

## Results

### Application User Interface

#### Sample Size Estimation

When designing a CRC study, it is important to collect enough data to achieve sufficient precision for PSE. *shinyrecap*'s sample size estimation tool does this by allowing the user to specify population parameters such as guesstimates of the target population size and the amount of capture heterogeneity in the population, as well as sample characteristics such as the number of capture events and their expected sample sizes. It then simulates CRC studies in this population and estimates the population size using log-linear modeling for each of the simulations. Precision is estimated from the simulation results. The application supports simulation and estimation using the M_t_ model if homogeneity is assumed. If heterogeneity is allowed, simulation and estimation are performed using the M_th_ model with normally distributed capture probabilities.

Given the input parameters, the interface provides the user with the distribution of a log-linear population size estimator across the simulations. A table is also provided that summarizes the percent of times simulated estimates were within different ranges of accuracy. A user might find it acceptable to have their estimate within 10% of the true value 90% of the time, whereas they might choose to collect more samples if the calculator says that their estimate will only be within 10% of the true value 50% of the time.

#### Data Formatting

The first barrier encountered by a practitioner is putting their CRC data into the right format for analysis. *shinyrecap* is able to read 2 data types: individual and aggregate. We focus on the capture history format (aggregate data) here to demonstrate the data formatter tool. Individual-level data files have 1 row per encounter, with each column representing a sampling event (eg, 3 columns for 3S-CRC) and, within the columns, the successful encounter event result (ie, the individual accepts the unique object; individuals who refuse the object during the encounter are not counted). The usual data format used by CRC analysis programs is the capture-history format. In this format, each column should represent a successful encounter event, and each row should be an encounter history. A “1” indicates a successful encounter (capture), and “0” indicates absence, so the following history represents 80 individuals who were encountered and accepted the unique object during the 2nd events, but not during the 1st or 3rd:



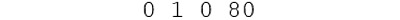



**Figure 1:**
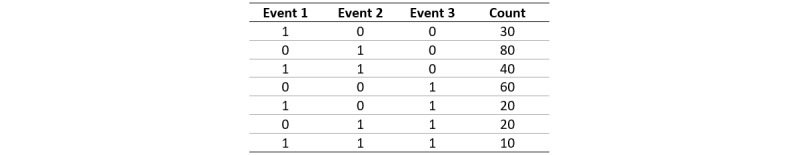
Example capture-history data format for 3 encounter events (3S-CRC). Absence or presence is denoted by 0 or 1, respectively.

When the aggregate data type is specified, the last column represents the total number of individuals with that capture history. A properly structured 3S-CRC data set would look something like Figure 1.

From the first row, we see that there were 30 individuals who were observed at event 1 but not at the 2nd and 3rd events:



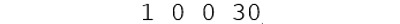



There were 10 individuals captured in all 3 events, as seen in the following row:



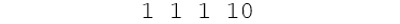



Note that there is no row for the following history because that pertains to the unknown number of population members who were not observed at any event:



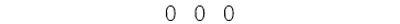



For *k* encounter events, there are 2*^k^* – 1 observable event histories and 1 unobservable history. Analysis of CRC data requires enumeration of all 2*^k^* – 1 observable counts (which may contain observed values of 0 but not missing values).

**Figure 2:**
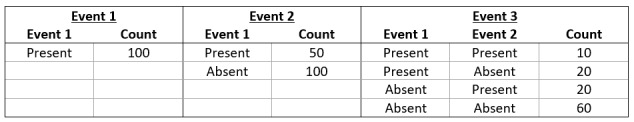
Aggregated capture histories in event-count format for 3-source capture-recapture (3S-CRC).

The capture-history data format is easily recorded from individually identifiable population members. However, in many epidemiological studies, unique individuals are not identified; rather, data are aggregated. These accumulated data files consist of counts of individuals who were encountered at each sampling event and the subsets of those who were encountered at any *preceding* sampling event(s) (Figure 2). No identifying information is collected on any subject at any event. During the 1st sampling event, only the count of individuals present and who were offered and accepted a unique (to the event) identifier is recorded. During the 2nd event, observed population members are tabulated by whether they received the identifier distributed during the first event, and those individuals are given a second (and different) aggregate identifier. At the 3rd event, the observed population members are cross-tabulated by whether they received the event-specific identifier distributed during each of the 2 previous events. We call this event-count formatted data. Although 7 counts have been recorded, the counts are aggregated differently from the required format shown in Figure 1. Note that the sum of samples should always be larger than the sum of count data.

It takes some thought to figure out how to convert the data to the required format, and the process becomes much more difficult if there are more than 3 events. The *shinyrecap* data formatting tool makes that conversion easy and reliable for any number of encounter events.

### Analysis

*shinyrecap* guides the user through the analysis process for log-linear modeling, Bayesian model averaging, and Bayesian latent-class modeling. All analyses may be exported as downloadable reports in HTML, Word, or PDF documents. To facilitate analysis transparency and reproducibility, R code to replicate the analysis is included in all reports by default.

#### Log-Linear Models

The log-linear section of the application has 3 sections. The first section, “Model Comparison,” displays population size, standard error, AIC, and BIC for each potential model formulation. The “Model Selection” section allows the user to select one of these models and calculate a confidence interval. The “Descriptives” section provides output to help the user understand the model and diagnose potential problems. Two diagnostic plots help explore the heterogeneity structure. The first diagnostic plot displays a function of the number of units captured *i* times for different values of *i*. It should be roughly linear except in the case where the data were generated by an M_th_ model. The second diagnostic plot shows the number of individuals captured for the first time at the *i*_th_ sampling event. It should be linear in the case of the M_0_ model and concave down in the case of an M_h_ model. Any other form may indicate an M_t_ or M_th_ model.

#### Bayesian Model Averaging

The first step in the Bayesian model averaging interface is to set a prior distribution for the population size. This is set to a noninformative 1/N distribution, but it is recommended to change this to something relevant to the population under study. To do this, the user can specify their beliefs for the median population size (ie, they believe that there is a 50% chance the population size is above it and 50% chance it is below) and the 90th percentile (ie, there is only a 10% chance the true population size is above this value). The application then parameterizes these beliefs as a log-normal distribution. The user may also specify a maximum population size to put an upper bound on the prior.

Once the prior is set, the user can go to the “Posterior Population Size” tab to obtain posterior estimates and credible intervals. The “Posterior Model Probabilities” tab allows the user to explore the different individual models that are averaged together and see their influence on the posterior.

#### Bayesian Nonparametric Latent-Class Models

The Bayesian nonparametric latent-class model is the most computationally intense analysis method. The user may control the Gamma distribution stick-breaking prior as well as set the maximum number of latent groups. Increasing the number of latent groups increases the computation time but, since the number of groups is determined by the algorithm, does not affect the results so long as it is set sufficiently large. Although 10 is a good default value, the user can increase that to ensure that this limit does not affect the results.

There is a number of MCMC sampling options available to the user. There are 2 primary considerations that the user should be aware of. First, the MCMC process must be at equilibrium. To ensure this, the first samples generated by the algorithm should be thrown out. The number of samples thrown out is controlled by the “burn-in” option. If there are any trends in the trace plot (available in the Diagnostics tab), the burn-in period may need to be increased. Second, the sample size must be large enough that the posterior is not dominated by sampling noise. With MCMC sampling, each sample is correlated with the last sample, so the effective sample size (also in the Diagnostics tab) is often much lower than the raw number of samples generated by the process. Typically, the user should aim for an effective sample size greater than 1000. The effective sample size can be increased by increasing the number of samples generated or the number of MCMC steps taken between samples, which reduces correlation.

After specifying the prior on the number of strata and the MCMC sampling parameters, a sample from the posterior distribution is produced by pressing the “Run” button. A progress bar displays the progress of each computational operation. A posterior summary will be displayed.

#### Pairwise Analysis

The pairwise analysis table displays PSE, standard error, and 95% confidence limits for each possible pair of sampling events and is used as a diagnostic step to examine sampling events for homogeneity. Similar PSE across pairwise results indicate the independence assumption may have been met, whereas differences across results suggest violations of the assumption. Any of the models available in the *shinyrecap* Analysis tool may be used to incorporate such dependence into models.

### Example With FSW Data

Estimates of key population size are critical for HIV program planning. For this reason, a large 3S-CRC activity was implemented in a subnational unit (SNU) of a sub-Saharan African country with high HIV burden and unmet need for HIV/AIDS treatment services. Using 3S-CRC data collected from FSW, we demonstrated the utility of the *shinyrecap* tool to estimate sample size sufficient for precision, format our data in preparation for analysis, and produce PSE using several different statistical models.

Between October 2018 and December 2018, 544 FSW hotspots in the SNU were sampled, representing 13,344 encounters with FSW over 3 sampling rounds. During encounters with FSW in hotspots, FSW distribution teams offered inexpensive and memorable objects that were unique to each of the 3 capture rounds. Eligible FSW who consented were considered enrolled in this PSE activity. In subsequent rounds, 1 week to 2 weeks apart, FSW were asked to show or describe objects they had received during previous rounds, and affirmative responses were tallied upon correct identification of the objects. Distributors recorded information on tablets and uploaded to a secure central server after each encounter. Data were aggregated into a table similar to Figure 1 for analysis.

In the following sections, we work through how the *shinyrecap* application was used to assist in the planning, data management, and analysis of this study.

### Sample Size Estimation

**Figure 3:**
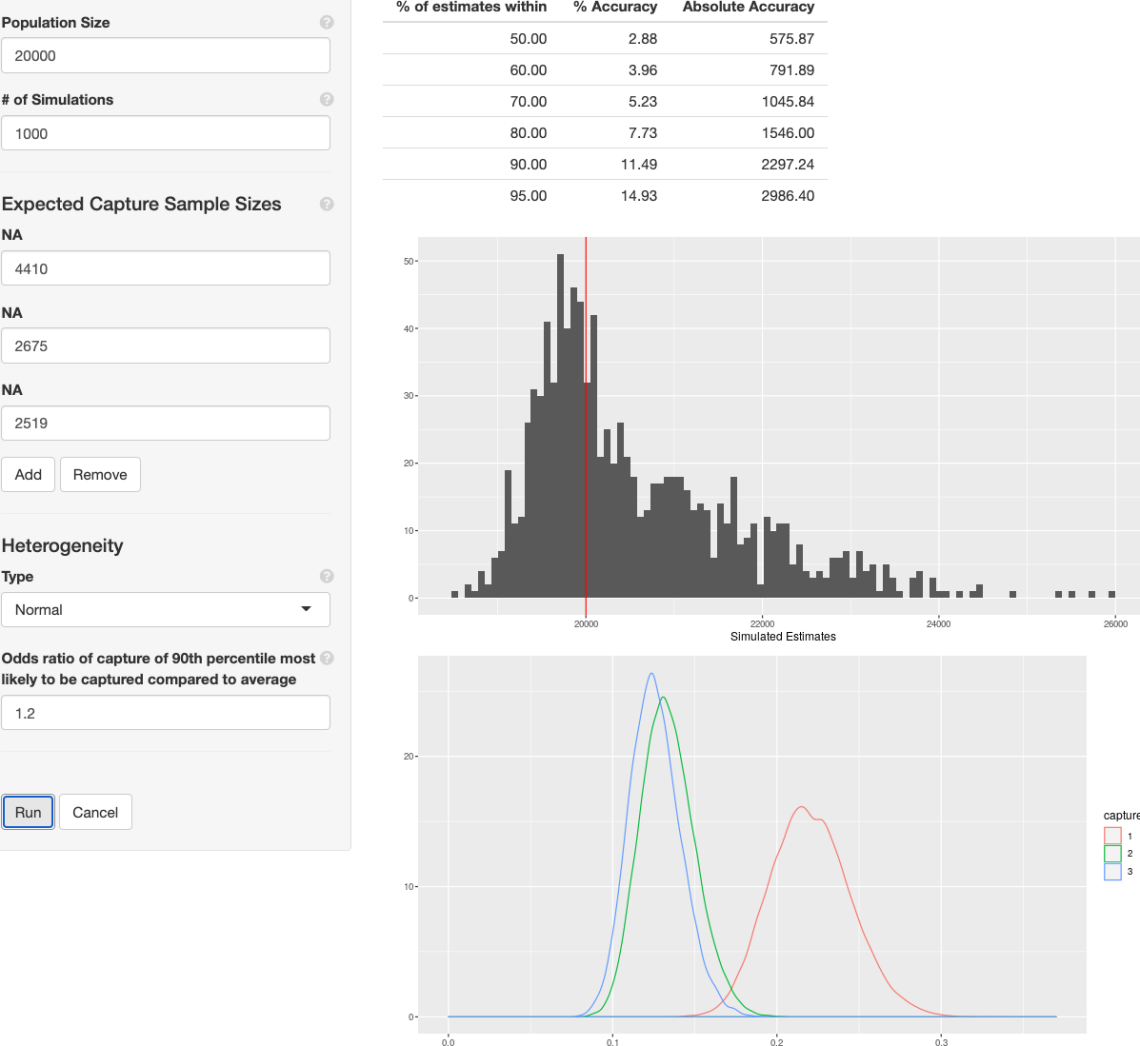
The sample size estimation tool applied to the example female sex worker study.

Before any study is conducted, it is wise to determine what level of precision one is likely to get out of a potential sampling plan. Figure 3 shows the result of using the sample size estimation tool in the context of the example FSW PSE study. Capture sample sizes were set at 4410, 2675, and 2519, with a theorized population size of 20,000. A moderate amount of heterogeneity was also added, such that 90% of individuals in the population had capture odds less than 1.2 times the average individual in the population.

The table in the upper right of Figure 3 summarizes the results and finds that, 80% of the time, our PSE will be within 7.73% of the true value.

### Data Format

**Figure 4:**
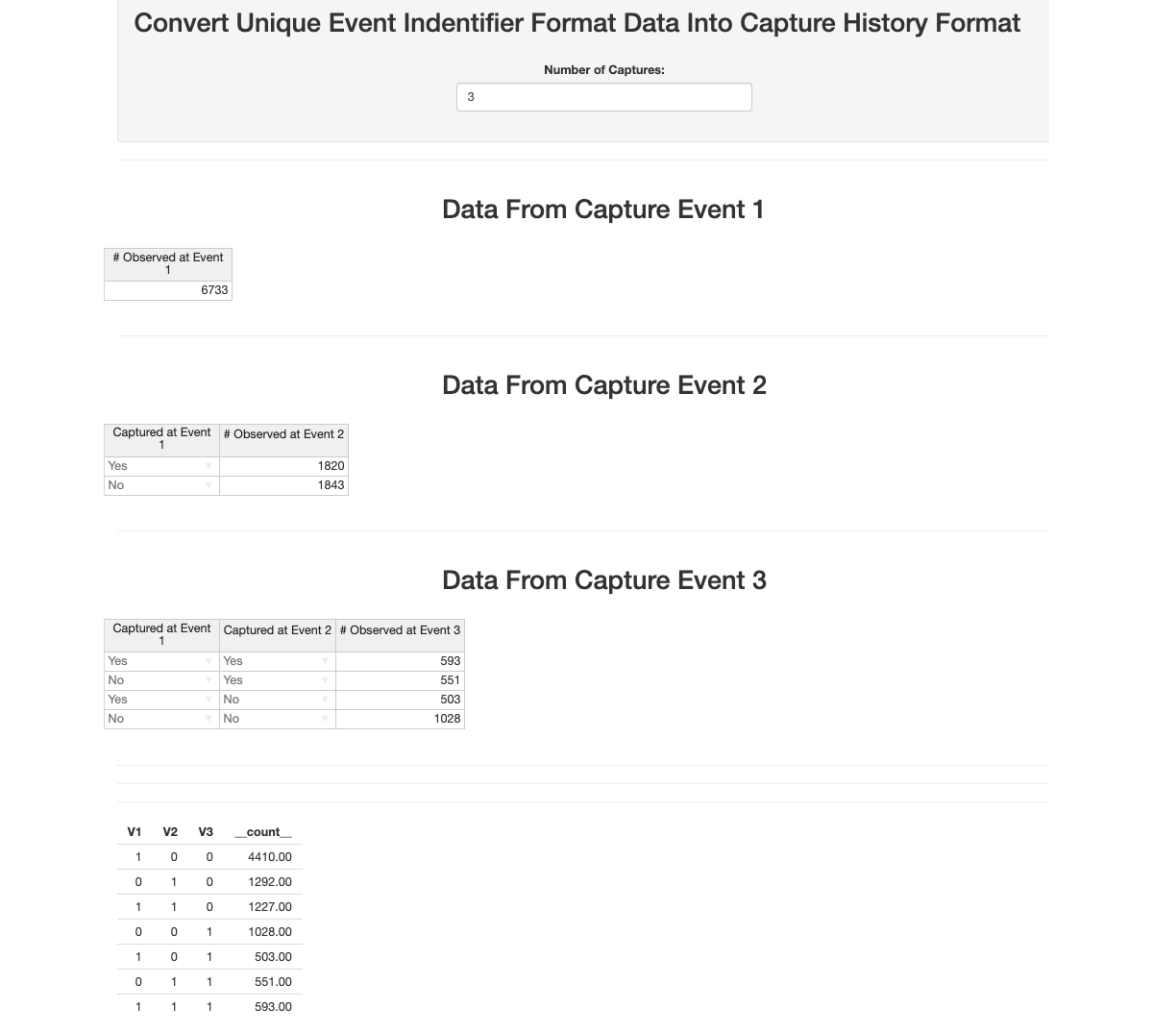
The data formatter tool.

After the data were collected, we translated it from event format to capture history format. Figure 4 shows the result of applying the data formatter to the example FSW CRC data. Once translated, the capture history data may be imported into the analysis tool for inference.

### Analysis

#### Log-linear Models

**Figure 5:**
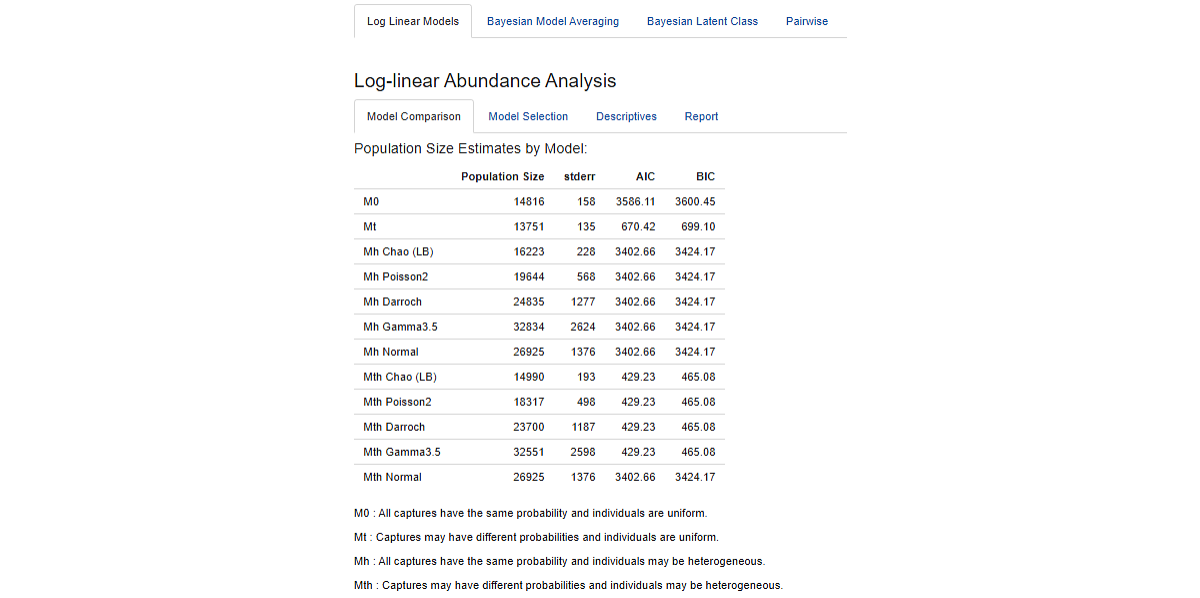
Log-linear models applied to the example female sex worker study.

The first class of models we apply is log-linear. Figure 5 displays the analysis tool’s result table for all of the various applicable log-linear models. These may have no effects (0), effects for time (t), effects for heterogeneity (h), or both (th). Note that there are multiple listings in the figure for heterogeneous models (h and th) corresponding to different functional forms for the differing capture probabilities of individuals in the population. For most epidemiological studies, we expect capture probability to vary among individuals or over time, which means that models M_t_ and M_th_ are likely more appropriate than the simpler alternatives. This is consistent with the result that the AIC and BIC values are considerably lower for these compared with the M_0_ and M_h_ models. The set of M_th_ models has the lowest AIC and BIC, indicating that there may be heterogeneity in the population.

Poisson2 induces a reasonable amount of heterogeneity and is generally a good default choice. In this case, it yields a PSE of 18,317, which, as we will see in the following sections, is consistent with other analyses.

#### Bayesian Model Averaging

**Figure 6:**
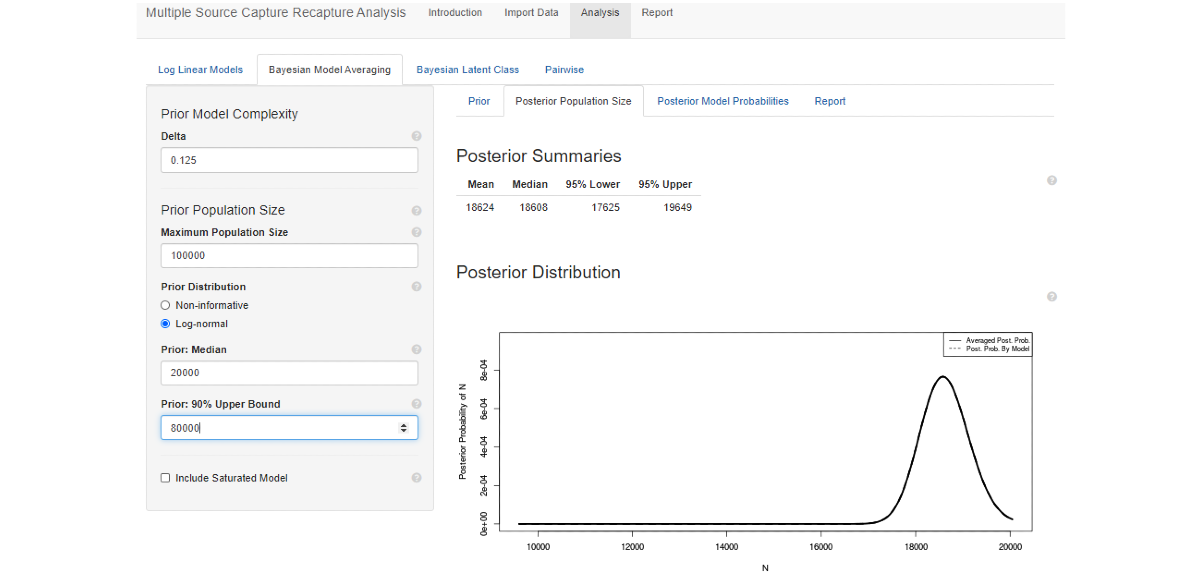
Bayesian model averaging applied to the example female sex worker study.

Applying a Bayesian model averaging model results in a very similar estimate compared with the Poisson2 log-linear model with a posterior estimate of 18,624 (see Figure 6). Here, we choose a diffuse prior for our analysis with a median population size of 20,000 and a 90th percentile of 80,000.

Use of the default “Noninformative” prior, which is an improper prior with mass equal to the inverse of population size, is a useful robustness check to assess the influence of our choice of prior. The posterior estimate using the noninformative prior was 18,608, which is very similar to our original result. Note that the log-normal prior median input was increased from the default of 7000 to 20,000 and the 90% upper bound was adjusted from the default of 10,000 to 80,000.

#### Bayesian Nonparametric Latent-Class Models

**Figure 7:**
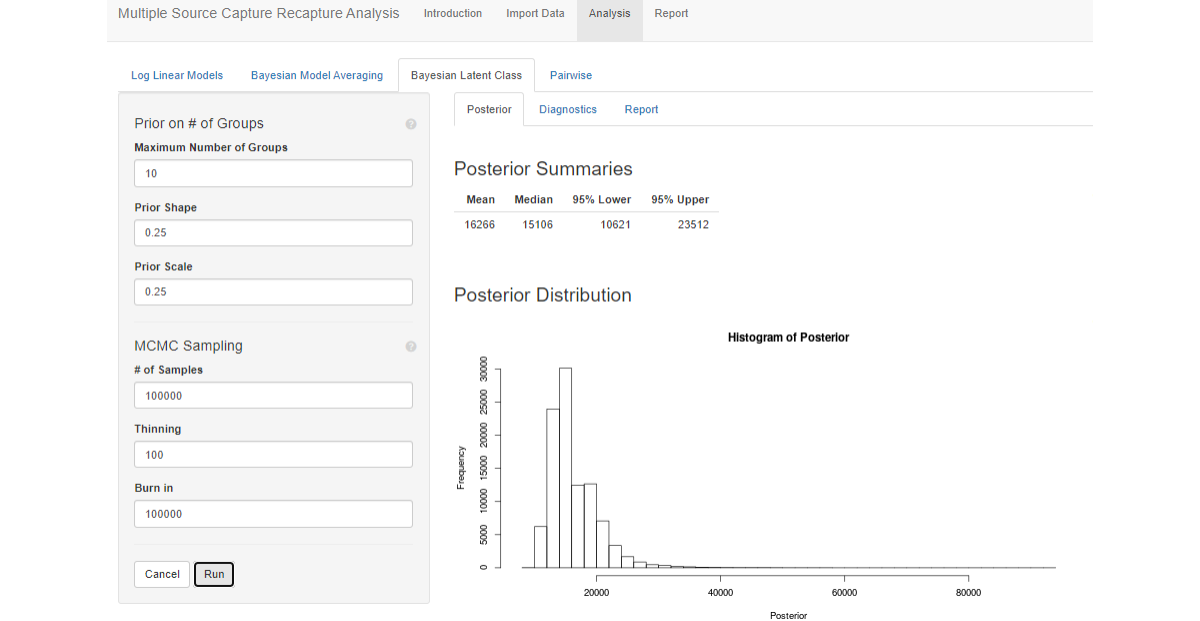
Bayesian latent-class modeling applied to the example female sex worker study.

Applying the latent-class model, as in Figure 7, results in an estimate of 16,266. This is modestly lower than the other methods; however, the 95% probability interval using this method is quite wide, ranging from 10,621 to 23,512, indicating that the model’s results are compatible with the other 2 methods. The latent-class model will often have intervals wider than the other 2 methods as a result of its high level of flexibility in describing the latent heterogeneity.

Note that the MCMC number of samples was increased to 100,000 from the default of 10,000, thinning was increased from the default of 10 to 100, and the burn-in was increased from the default of 10,000 to 100,000. These inputs were adjusted to increase effective sample size.

#### Pairwise Comparison

**Figure 8:**
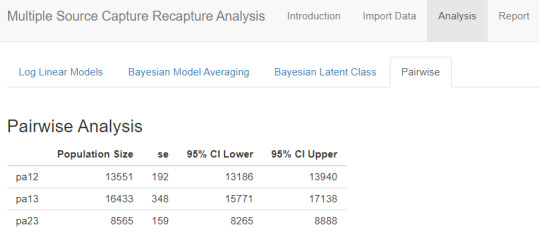
Pairwise analysis of example female sex worker study results.

The table in Figure 8 displays population estimates using each pair of capture events. This pairwise analysis may be helpful to review as a diagnostic step to understand the 3S-CRC data. Each row is a separate 2S-CRC analysis using only 2 of the sampling events. For example, pa12 estimates the population size using only the 1st and 2nd sampling events, pa13 estimates only the 1st and 3rd sampling events, and pa23 estimates only the 2nd and 3rd sampling events. The ideal situation is to have similar PSE for each pair, which would be consistent with independence of sampling events. Neither the 1st to 2nd nor the 1st to 3rd comparisons have intervals that overlap with the interval for the 2nd to 3rd comparison, suggesting that the independence assumption may be unreasonable for these.

## Discussion

*shinyrecap* is a new Shiny application for population size estimation that is easy to use and freely accessible to anyone with an internet connection and a web browser.

The example using 3S-CRC data from FSW in an SNU of a sub-Saharan African country demonstrates how computationally intensive statistical methods are made more accessible to epidemiologists and others with *shinyrecap*. The simplicity of the sample size estimation, data formatting, and analytic tools, with supporting online manuals and tutorial videos, allows users to progress through CRC activities when statistical support might not be readily available. *shinyrecap* promotes local ownership of PSE activities, including sample size determination, formatting data for use in the *shinyrecap*, as well as using the various analytical models for estimating population size. With several key statistical models available to those without coding expertise, local public health staff were able to test various models, compare the results, and interpret the results given their local knowledge.

Our model results using *shinyrecap* with 3S-CRC data were larger than the PSE produced from programmatic mapping and enumeration among FSW in the same SNU: 9858 in 2013 [43] and 9745 in 2015 [44]. Both these estimates were smaller than those produced by *shinyrecap*: log-linear models; for example, the M_th_ Chao lower bound was the smallest of all log-linear models, at 14,990 (14,620-15,378); the Bayesian model averaged 18,624 (17,625-19,649); and Bayesian latent-class models averaged 16,266 (10,612-23,512). The ability to produce confidence bounds is another benefit of *shinyrecap* compared with programmatic mapping and enumeration.

Shiny apps offer a solution to the problem of poor-quality estimates for key population program and policy developers and elevate the level of sophistication of analysis while building in-country capacity to implement critical surveillance activities. Recently, several Shiny apps were introduced that enhance HIV surveillance efforts to estimate awareness of HIV status over time [45], synthesize multiple PSE using the Anchored Multiplier [46], and estimate sample size for biobehavioral survey-based multiplier methods for PSE [47]. *shinyrecap* is unique among this group in that it offers multiple features in one tool to support population size estimation with 3S-CRC from sample size estimation to data formatting to multiple model options for analysis.

Our work was motivated by the needs of epidemiologists and others who require reliable tools for PSE but may not have the necessary coding experience or advanced statistical skills needed to analyze CRC data involving 3 or more samples. The application facilitates the estimation of sample sizes for captures, proper formatting of individual-level and aggregate-level data in preparation for analysis, and various options for analysis of CRC data from 3 or more sources. In addition, all output can be saved in HTML, Word, or PDF formats, with an option to include the R code used by the Shiny to produce the output. Public health teams now have a powerful tool in *shinyrecap* to produce reliable PSE for a broad range of applications without specialized computing expertise.

## Abbreviations

2S-CRC: 2-sample capture-recapture
3S-CRC: 3 (or more)-sample capture-recapture
AIC: Akaike information criterion
BIC: Bayesian information criterion
CRC: capture-recapture
FSW: female sex worker
MCMC: Markov chain Monte Carlo
MSM: men who have sex with men
PSE: population size estimate
PWID: people who inject drugs
SNU: subnational unit
SSL: secure-sockets layer
